# Supplementary material for: What is the impact of long-term COVID-19 on workers in healthcare settings? A rapid systematic review of current evidence
Source: PLoS One. 2024 Mar 5;19(3):e0299743. doi: 10.1371/journal.pone.0299743 (PMC10914278; doi:10.1371/journal.pone.0299743)
Supplement: S1 Appendix — (DOCX) [file pone.0299743.s001.docx]

# Appendix 1 Medline search strategy

1 exp *Health Personnel/

2 ((health care or healthcare or medical or clinic* or hospital? or health service* or care or nursing or general practice or family practice or mental health or psychiatric or psychology or outpatient or pharmacy or dental or community or intermediate care or rehabilitation or ancillary or domestic or estates or non-medical or non-clinical or administrat* or support) adj3 (worker* or professional* or staff or practitioner* or employee* or personnel or assistant or workforce or student)).tw,kf.

3 (doctor? or physician? or clinician? or paramedic? or nurse? or surgeon? or consultant? or therapist? or practitioner? or radiographer? or dieti* or physiotherapist? or chiropodist? or podiatrist? or orthoptist? or osteopath? or paramedic? or prosthetist? or orthotist? or psychologist? or psychiatrist? or scientist? or "health visitor?" or midwi#e).tw,kf.

4 1 or 2 or 3

5 (((long or chronic or persist* or residual or post or postacute or postinfec* or postvir* or relaps*) adj3 (covid* or coronavirus* or Cov or "SARS-CoV-2*" or "SARSCoV-2*" or "SARSCoV2*" or "SARS-CoV2*")) or long-haulers or postcovid*).tw,kf.

6 4 and 5
