# Supplementary material for: What is the impact of long-term COVID-19 on workers in healthcare settings? A rapid systematic review of current evidence
Source: PLoS One. 2024 Mar 5;19(3):e0299743. doi: 10.1371/journal.pone.0299743 (PMC10914278; doi:10.1371/journal.pone.0299743)
Supplement: S2 Table — (DOCX) [file pone.0299743.s003.docx]

| **Study ID (country)** | **Methods for data collection and analysis** | **Study objectives** | **Sample size and participant characteristics** | **Summary of main results** |
| --- | --- | --- | --- | --- |
| **Brandt 2021^19^ (Germany)** | Interviews with staff members six months after testing positive for COVID. Descriptive statistics used  Follow-up: 6 months after infection | Report of a COVID outbreak in a hospital department | Total n=13  Occupation: NR  Age (range): 38.2 (23-55)  Sex: 6 male (46.2%); 7 female (53.8%) | 3/13 (23.1%) had long-term symptoms:   - Anosmia, ageusia & dysgeusia: 1/13 (7.7%) - Paraesthesia: 1/13 (7.7%) - Weakness/numbness: 1/13 (7.7%) |
| **Gaber 2021^22^ (UK)** | Questionnaires were sent by email. Descriptive statistics used  Follow-up: NR | Investigation of the long-term impact of COVID-19 on healthcare workers | Total n=138  Occupation: NR  Age: NR  Sex: 11 male (8.0%); 127 female (92.0%) | 44.2% had long-term symptoms:   - Sleep problems: 48.6% - Mood disorders: 44.2% - Shortness of breath: 39.9% - Fatigue: 39.1% |
| **Havervall 2021^23^ (Sweden)** | Longitudinal cohort study. Follow-up at 8 months by smartphone app. The analysis included associations between categorical variables using the χ2 test of independence. Risk ratios (RRs) and 95%CIs calculated comparing seropositive and seronegative participants for moderate to severe symptoms lasting 2 or more or 8 or more months and for moderate to marked disruption on the Sheehan Disability Scale  Follow up: >8 months after infection | Investigation of COVID-19-related long-term symptoms in healthcare professionals | Total n=1395 (323 seropositive and 1072 seronegative)  Occupation: NR  Age, median (IQR):   - Seropositive: 43 (33-52) - Seronegative: 47 (36-56)   Sex:   - Seropositive:   - 55 male (17.0%)   - 268 female (83.0%) - Seronegative:   - 147 male (13.7%) 925   - female (86.3%) | 14.9% seropositive/ 3.4% seronegative had symptoms after 8 months:   - Anosmia:   - 9.0% seropositive   - 0.1% seronegative - Fatigue:   - 4.0% seropositive   - 1.5% seronegative - Ageusia:   - 3.7% seropositive   - 0.1% seronegative) |
| **Martinez 2021**  **(Switzerland)** | Retrospective cohort study; online questionnaire of HCWs with confirmed COVID-19 infection.  Uni- and multivariable logistic regression (using stepwise selection) to test association between baseline characteristics and symptom persistence  Follow-up: 90 days after COVID-19 diagnosis | To assess the frequency of persisting symptoms after COVID-19 infection in HCWs at a university  hospital in Switzerland | Total n=260  Occupation: 123 nursing staff (47.3%)  Age: <30, 74 (28.5%); 30-49.99, 122 (46.9); >=50, 64 (24.6%)  Sex: 196 female (75.4%), 64 male (24.6%) | 69/260 (26.5%) reported not having regained their usual level of health or having had a symptom duration of more than 3 months. Most commonly reported symptoms were fatigue (68.9%), impaired taste or smell (51.1%), and 1412 cumulative missed workdays (median 15, IQR  10–21). |
| **Mattioli 2021^24^ (Italy)** | Healthcare workers previously infected with COVID-19 were examined 4 months after diagnosis. Descriptive statistics presented plus comparisons of people positive and negative for COVID using Student’s T-test, ANOVA, or the Mann–Whitney and Kruskal–Wallis test; correlations among variables by the Pearson’s or Spearman’s rank correlation test  Follow-up: 4 months | Investigate the presence of neurological focal deficits and/or cognitive impairment in healthcare workers 4 months after diagnosis | Total n=120  Occupation: 20 doctors & biologists (16.7%); 71 nurses, physiotherapists & technicians (59.2%); 29 health auxiliaries (24.2%)  Age, years, median (range): 47.9 (26-65)  Sex: 30 male (25.0%), 90 female (75.0%) | 65% had long-term symptoms:   - Anosmia: 19.2% - Fatigue: 15% - Headache: 12.5% - Attention difficulties: 11.7% - Ageusia: 10.8% - Dyspnoea: 10.8% - Joint/muscle pain 9.2% - Insomnia 6.7% - Memory difficulties 6.7% - Irritability/ anxiety 5% - All other symptoms in <5% participants |
| **Nielsen 2021^25^ (Denmark)** | Baseline questionnaire followed by daily text/email reporting of symptoms. Odds ratios for the seven specific symptoms were estimated by test results (positive, negative) for three time periods  Follow-up: 90 days after being tested | Comparison of symptoms day-by-day for non-hospitalised healthcare workers testing positive and negative for COVID-19 | Total n=840 (210 tested positive, 630 tested negative)  Occupation:  Positive test:  140 nursing staff (66.7%); 38 medical doctors (18.1%); 8 biomedical laboratory scientists (3.8%); 5 medical secretaries (2.4%); 19 other (admin, service & technical staff) (9.0%)  Negative test:  290 nursing staff (46.0%); 111 medical doctors (17.6%); 37 biomedical laboratory scientists (5.9%); 39 medical secretaries (6.2%); 153 other (admin, service & technical staff) (24.3%)  Age, n (%):  Positive test:  <30y, 33 (15.7%); 30-39y: 49 (23.3%); 40-49y: 64 (30.5%); 50-59y: 49 (23.3%); >=60y: 15 (7.1%)  Negative test:  <30y, 58 (9.2%); 30-39y: 153 (24.3%); 40-49y: 221 (35.1%); 50-59y: 146 (23.2%); >=60y: 52 (8.3%)  Sex:  Positive test: 33 male (15.7%); 177 female (84.3%)  Negative test: 99 male (15.7%) 531 female (84.3%) female | Of those who tested positive, 44.1%/38.5% had symptoms at days 31-60 and 61-90, respectively:   - Reduced/lost taste/smell:   - Days 31-60: 29.3%   - Days 61-90: 28.6% - Headache:   - Days 31-60: 8.8%   - Days 61-90: 6.6% - Cough:   - Days 31-60: 10.6%   - Days 61-90: 4.1% - Dyspnoea:   - Days 31-60: 4.7%   - Days 61-90: 3.5% |
| **Pereira 2021^26^ (UK)** | Descriptive study of healthcare workers. Follow-up questionnaire to assess symptoms completed 7 to 8 months after symptom onset. Statistical tests used to compare physiological parameters and the likelihood of PCS  Follow-up: 7 to 8 months after symptom onset | Association between antibody response to COVID-19 and post-COVID-19 syndrome in healthcare workers | Total n=38 (21 post-COVID-19 syndrome, 17 non-post-COVID-19 syndrome)  Occupation:  Post-COVID-19 syndrome: 6 administrators (28.6%), 1 dietician (4.8%), 2 housekeeping staff (9.5%), 1 physician (4.8%), 6 nursing staff (28.6%), 3 OT or physio (14.3%), 2 pharmacists (9.5%)  Non-post-COVID-19 syndrome: 2 administrators (11.8%), 1 housekeeping staff (5.9%), 3 physicians (17.6%), 6 nursing staff (35.3%), 4 OT or physio (23.5%), 1 phlebotomist (5.9%)  Age, mean, years: Post-COVID-19 syndrome: 43; Non-post-COVID-19 syndrome 44  Sex, n (%):  Post-COVID-19 syndrome: 1 male (4.8%), 20 female (95.2%)  Non-post-COVID-19 syndrome: 5 male (29.4%), 12 female (70.6%) | 55.3% had long-term symptoms:   - Fatigue: 57% - Loss of smell: 29% - Breathlessness: 24% - Difficulty concentrating: 24% |
| **Rao 2021^27^ (India)** | Web-based single centre survey study. Descriptive statistics used  Follow-up: NR | Assessment of health, social and psychological impact on HCWs who had recovered from COVID-19 | Total n=163  Occupation: 51% doctors; 31% nurses; 9% AHP; 9% students  Age, years, %: <30: 52%, 30-40: 32%, 40-50: 11%, 50-60: 3%,  >60: 1%  Sex: 41% male, 59% female | Proportion with long-term symptoms NR:   - Fatigue: 42.9% - Loss of taste/smell: 21.5% - Headache: 15.3% - Breathlessness: 8.6% - No symptoms: 33.7% |
| **Sultana 2021^28^ (Bangladesh)** | Descriptive cross-sectional study. Descriptive statistics and logistic regression model of the relationship between Long COVID symptoms and explanatory variables  Follow-up: >60 days since infection; mean 124 days | Estimate prevalence, length of illness, and risk factors of post-COVID symptoms to highlight the burden of the disease for HCW and recognise those at higher risk | Total n=186  Occupation: 186 doctors (100%)  Age, mean (SD), years:34.8 (9.9)  Sex: 123 male (66.1%), 63 female (33.9%) | 69.9%/23.7% had long-term symptoms at ≤60 days/>60 days, respectively:   - Fatigue   - ≤60 days: 5.4%   - >60 days: 8.1% - Difficulty breathing   - ≤60 days: 2.2%   - >60 days: 6.5% - All other symptoms in <5% participants at both time points |
| **Tawfik 2021^29^ (Egypt)** | Retrospective cohort study. Descriptive statistics and odds ratios analysed  Follow-up: 1 month and >3 months | Detect post-COVID-19 syndrome in HCW | Total n=120  Occupation: Physicians, nurses, dentists and pharmacists (no further details reported)  Age, mean (SD), years: 33.7 (7.3)  Sex; 50 male (42%), 70 female (58%) | Proportion with long-term symptoms NR:   - Fatigue:   - 1 month: 75%   - 3 months: 33% - Dyspnoea   - 1 month: 50%   - 3 months: 29% - Depression:   - 1 month: 50%   - 3 months: 20% - Headache:   - 1 month: 42%   - 3 months: 19%   Note: above numbers are approximate |
| **Tempany 2021^20^ (Ireland)** | Survey of HCW who attended for SARS-CoV-2 antibody testing. Descriptive statistics and chi-square tests to compare differences in known and assumed infection groups  Follow-up: ≥12 weeks | Assessment of prevalence of Long COVID symptoms and the subjective degree of recovery in HCW who have returned to work | Total n=217 (139 known infection, 78 assumed infections)  Occupation: NR  Age, range, years: 20-69  Sex, n (%):  Known infection: 28 male (20.1%), 111 female (79.9%)  Assumed infection: 15 male (19.2%), 63 female (80.8%) | 70.5% had long-term symptoms:   - Fatigue: 56.1% - Sleep disturbance: 40.3% - Cognitive impairment: 24.5% - Psychological symptoms: 21.6% |
| **Akova 2022 (Turkey)** | Survey of HCW with Long COVID who attended an out-patient clinic for any reason between 01-03-21 and 15-04-21. Descriptive statistics and binary logistic regression to predict fatigue scores and sleep quality  Follow-up: NR | To explore the fatigue and sleep quality of HCW with Long COVID and their predictors | Total n=133  Occupation: 45 physicians (33.8%), 36 nurses/midwives (27.1%), 52 other HCW (39.1%)  Age, mean (SD), years: 36.0 (9.7)  Sex: 60 male (45.1%), 73 female (54.9%) | 55.6% were fatigued/over- fatigued (predicted by being female, being overweight or by fatigue score)  59.4% reported poor sleep quality (significantly more females than males) |
| **Carazo 2022 (Canada)** | Case-control survey-based study comparing symptoms lasing ≥4 weeks in symptomatic HCWs and negative controls. Four-weekly prevalence over time assessed using Cochran-Armitage test. Spearman correlation coefficient used to assess correlation between symptoms  Follow-up: NR | Estimation of (1) prevalence and risk factors for persistent COVID-19 symptoms 5-28 weeks after acute illness among predominantly non-hospitalised HCWs and (2) contribution of Long-COVID, fatigue and psychological distress on persistent neurocognitive symptoms | Total n=6061 COVID-19 cases at 4 weeks, 1783 COVID-19 cases at 12 weeks and 4390 controls  Occupation: COVID-19 cases, 243 physicians (4.0%), 1138 nurses (18.8%), 484 nurse assistants (8.0%), 1566 healthcare assistants (25.9%), 202 housekeepers (3.3%), 604 administrators/managers (10.0%), 202 psychosocial workers (3.3%), 1603 other (26.5%)  Age, mean (SD), years: hospitalised HCW 46.7 (11.9); non-hospitalised HCW 40.0 (12.1); controls 39.0 (10.4)  Sex: hospitalised HCW, 35 male (29.7%), 83 female (70.3%); non-hospitalised HCW, 1230 male (20.7%), 4704 female (79.3%) | 46.2% of the non-hospitalised COVID-19 cases reported symptoms persisting at least 4 weeks and 39.9% with symptoms lasting at least 12 weeks. Of those hospitalised with COVID-19, 76.3% and 67.6% reported symptoms persisting at least 4 and 12 weeks, respectively. The most commonly reported symptoms lasting at least 4 weeks were fatigue, loss of smell/taste, shortness of breath, cognitive dysfunction, headache and joint/muscular pain. Self-reported cognitive dysfunction was 2 to 3 times more frequent in COVID-19 cases than controls |
| **Kameyama 2022**  **(Japan)** | Anonymous questionnaire to HCWs affected by a COVID-19 outbreak.  Statistical differences detected by Student t-test or Mann-Whitney U test  Follow-up: 6 months | To investigate  the physiological and psychological outcomes of HCWs infected with  COVID-19 during an outbreak in a hospital | Total n=83  Occupation: 10 doctors (12.0%), 52 nurses (62.7%), 7 nursing assistants (8.4%), 1 pharmacist (1.2%), 1 medical technologist (1.2%), 2 radiological technologists (2.4%), 5 rehabilitation technicians (6.0%), 3 dental hygienists (3.6%), 1 childcare worker (1.2%), 1 hospital clerk (1.2%)  Age, median (IQR): 34.0 (25.0-48.0)  Sex: 23 male (27.7%), 60 female (72.3%) | 3 months after infection, anosmia  (18.1%) was the most frequent symptom, followed by fatigue (9.6%) and headache (9.6%)  6 months after infection, the most common symptom was anosmia (7.2%) followed by fatigue (4.8%). Numbers not reported.  median EQ-VAS score was 75.0, and the median  motivation score was 4.0. |
|  |  |  |  |  |
| **Kaplan 2022**  **(Turkey)** | Questionnaire to HCWs who had previously tested positive for COVID-19. Categorical variables analysed by chi-square test. Risk factors determined by logistic regression  Follow-up: ≥12 weeks | To investigate persistent symptoms of HCWs after COVID-19 | Total n=121  Occupation: 63 doctors (52.1%), 30 nurses (24.8%), 28 other HCWs (23.1%)  Age, mean (SD): 33.5 (8.2)  Sex: 39 male (32.2%), 82 female (62.8%) | The most common symptoms for >3 weeks after COVID-19 infection were in 77 (63.6%) participants. Fatigue (n=40, 33%), loss of smell (n=27, 22.3%), attention deficit/concentration disorder (n=25, 20.7%), dyspnea (n=24, 19.8%), myalgia (n=24, 19.8%), loss of taste (n=23, 19%), cough (n=19, 15.7%), joint pain (n=18, 14.9%), sleep disturbance (n=14,  11.6%), and memory difficulties (n=13, 10.7%)  40 (33.1%) participants had worse health status than before |
| **Kinge 2022**  **(South Africa)** | An anonymous cross-sectional online survey of frontline HCW  Chi-square or Fisher’s exact tests to test differences between categorical variables. T-test and Mann-Whitney-U-test to compare scores  Follow-up: up to 1 year | To identify and determine the prevalence of  persistent/long COVID-19 symptoms among frontline workers | Total n=62  Occupation: NR  Age, median (IQR): 33.5 (30-44)  Sex: 15 male (24.2%), 47 (75.8%) female | Persistent (long) COVID-19 (symptoms experienced for three months and longer) was observed in 24.2% (15 out of 62) of the respondents; 33% of those experienced more than one symptom |
| **Mendola 2022**  **(Italy)** | Occupational health surveillance programme and structured questionnaire.  Kaplan-Meier method for survival analyses of patients with specific mild symptoms. Log-rank test was used to detect significant differences between groups  Follow-up: 18 months after acute infection | Describe the main long-term effects of COVID-19 infection among HCWs requiring hospitalisation and the subsequent implications regarding perceived work ability and fitness to work | Total n = 56  Occupation: 19 physicians (33.9%), 23 nurses (41.1%), 10 nursing assistants (17.9%), 4 other HCWs (3.6%)  Age: median (IQR), 55 (50-61.2)  Sex: 28 male (50.0%), 28 female (50.0%) | Post-COVID-19 symptoms most commonly reported:   - Exertional dyspnea (86.8%) - Asthenia (86.8%) - Arthromyalgia (71.7%) - Sleep disorders (64.2%) - Resting dyspnea (62.3%) - Cough (56.6%) |
| **Mohr 2022**  **(USA)** | Electronic surveys or interviews (online, by phone or in person) with follow-up survey at 6 weeks.  Multivariable Poisson regression, Poisson regression, Wilcoxon rank-sum test, Kaplan-Meier survival curves, log-rank test to test for risk differences and differences in times to return to work  Follow-up: 6 weeks | To compare the prevalence of symptoms 6 weeks after onset of COVID-19 illness among HCWs and days to return to work by vaccination status before their infection | Total n=419  (Vaccinated, n=180  Unvaccinated, n=239)  Occupation: 128 Non-clinical (30.5%), 20 physicians (4.8%), 12 advanced practice providers (2.9%), 164 nurse/nurse assistants (39.1%), 2 housekeeping (0.5%), 48 other clinical (11.5%), 45 other (10.7%)  Age group, n (%): 18-29, 90 (21.5); 30-39, 167 (39.9); 40-49, 85 (20.3); 50-64, 77 (18.4)  Sex: 64 male (15.3%), 352 female (84.0%), 1 non-binary (0.2%), 2 missing data (0.5%) | 298 (71%) reported one or more COVID-like symptoms 6 weeks after illness onset, 56.3% reported at least one neurological symptom and 75.9% reported any symptom.  Vaccinated HCP returned to work a median 2.0 days (95% CI 1.0 to 3.0) sooner than unvaccinated HCP (adjusted HR 1.37, 95% CI 1.04 to 1.79) |
| **Nehme 2022**  **(Switzerland)** | Online questionnaire to all HCWs (and non-HCWs tested for COVID-19)  Descriptive analyses and prevalence included percentages, with comparisons using chi-square tests or Fisher’s exact test.  To evaluate the effect of HCW versus non-HCW and SARS-CoV-2 infection on the outcomes a causal mediation analysis was conducted and multivariable logistic regression models.  Follow-up: median 244 days (IQR 202-400) | Differentiate the direct effects of COVID-19 from the pandemic-related indirect effects on HCWs at a Geneva hospital (and comparing results to non-HCWs) | Total: n=6639  (n=3083 HCWs [65.0 tested negative, 35.0% positive] and n=3556 general population)  Occupation all HCWs: 43.9 % nursing staff, 19.3 % administrative staff, and 15.9 % physicians  Age, years, mean (SD):  All HCWs: 43.8 (11.0)  General population: 44.4 (14.4)  Sex:  All HCWs: 2228 (72.3%) female (72.3%), 855 male (27.7%)  General population: 2009 female (56.5%), 1547 male (43.5%) | Presence of symptoms was greater in COVID-19 positive HCWs than negative HCWs |
| **Otmani 2022 (Morocco)** | Case-control survey study of HCW with COVID-19 and controls with no symptoms. Analysis NR  Follow-up: NR | Estimate prevalence, symptoms and signs extending beyond the scute phase of COVID-19 compared to the general population not infected with COVID-19 and to assess factors influencing the occurrence of symptoms | Total n=118 infected with COVID-19, n=118 not infected  Occupation: COVID-19 cases, 92 doctors (78%), 26 other HCW (22%)  Age, mean (range): infected, 29 (21-54), not infected, 29 (21-54)  Sex: infected, 34 male (29%), 84 female, not infected, 34 male (29%), 84 female (71%) | 56/118 (47.4%) experienced symptoms of Long COVID. Those who had been infected with COVID as compared to controls had a statistically significantly higher prevalence of asthenia (25.3%), “brain fog” (14.4%), myalgia (13.3%), headache (12%), sleep disorders (12%) and anosmia/hyposmia (9.6%). |
| **Pilmis 2022**  **(France)** | Study conducted at 894-bed tertiary-care teaching hospital in Paris, France employing 3612 HCWs. All HCWs with positive SARS-CoV-2 serology were assessed using a questionnaire and longitudinal follow-up  Follow-up: 7 months | Understand the immune response following SARS-CoV-2 infection and determine the infection prevalence among HCWs after the first wave of the pandemic | Total n=74 included in the 7-month cohort  Occupation: NR  Sex: 61 female (82.4%), 13 male (17.6%)  Age, median, years: 47 [IQR 33.2–54.2 years] | 24/74 (32.4%) experienced post-acute COVID-19 syndrome. Symptoms were were asthenia (16.2%) dyspnea (13.5%) and concentration disorder (9.5%). |
| **Selvaskandan 2022**  **(UK)** | Online questionnaire of nephrology affiliated HCWs including doctors, nurses, technicians, pharmacists, psychologists, and social workers.  Descriptive statistics with proportions. Comparisons of categorical variables with chi-squared test or Fisher’s exact test. Association examined with multivariable logistic regression models. Odds ratios with 95% confidence intervals reported  Follow-up: 12 months | Identify the impact of COVID-19 pandemic on the nephrology workforce, 12 months on, with regards to the incidence of COVID-19 and Long COVID and its impacts on working lives. | Total n=423 (120 with COVID-19 diagnosis)  Occupation: 144 doctors (34%), 150 nurses (36%), 125 other multidisciplinary professionals (30%), 1 retired (<1%)  Sex: 313 female (74%), 110 male (26%)  Age group:  <25 years, 2 (0.4%)  25–34 years, 63 (15%)  35–44 years, 110 (26%)  45–54 years, 151 (36%)  55–64 years, 91 (22%)  ≥65 years, 5 (1%) | 43/120 (36%) who had COVID-19 had persistent symptoms consistent with Long COVID  Most common symptoms were fatigue (70%), mood changes (19%) and ageusia/anosmia (14%). Impacts of Long COVID on quality of life and work were reported. Those with Long COVID were 10 times more likely to experience burnout and more likely to want to retire early |
| **Senjam 2022**  **(India)** | Cross-sectional questionnaire study of HCWs and their dependents. Descriptive analysis; Chi Square, Fisher’s exact test and t-test to compare variables; logistic regression models to explore associations with symptoms  Follow-up: 4 weeks | Comprehensively assess self-reported post COVID-19 symptoms and associated risk factors among beneficiaries of a Hospital Employee Scheme of a tertiary healthcare institution in Delhi | Total n=395 hospital employees  Occupation: NR  Age: NR  Sex: NR | 156/395 (39.5%) reported post-COVID symptoms (≥4 weeks) |
| **Strahm 2022**  **(Switzerland)** | Prospective questionnaire cohort study comparing Long COVID symptoms in HCWs with positive nasopharyngeal swab (NPS), HCWs with a- or pauci-symptomatic (i.e. only seropositive) COVID-19, and negative controls.  Analysis using Chi-squared tests, odds ratios, univariable and multivariable quasi-Poisson regression  Follow-up: median 117 days since acute infection | Assess the frequency  and risk factors for symptoms compatible with Long COVID | Total n=3334 (556 with positive NPS, 228 only seropositive, 2550 negative controls)  Occupation: Positive NPS, 329 nurses (59%), 70 physicians (13%), 141 other (25%); only seropositive, 131 nurses (57%), 25 physicians (11%), 59 other (26%); negative controls, 1033 nurses (41%), 428 physicians (17%), 980 other (38%)  Age, median (range): Positive NPS, 38.9 (16.8-63.5); only seropositive, 37.9 (17.1-63.9); negative controls, 41.0 (16.5-72.6)  Sex: Positive NPS, 98 male (18%), 453 female (81%); only seropositive, 45 male (20%), 182 female (80%); negative controls, 529 male (21%), 2003 female (79%) | HCW reporting ≥1 symptom was higher for HCW with positive NPS compared to negative controls (73% vs. 52%, p<0.001), but not for seropositive HCW  without positive NPS (58% vs. 52%, p=0.13).  The most common symptoms were exhaustion/burnout (33% in NPS-positive vs. 25% in only seropositive vs. 24% in negative controls) and weakness/tiredness (34% vs. 25% vs. 22%). Impaired  taste/olfaction (33% vs. 16% vs. 6%)  NPS-positive participants (but not seropositive participants without positive swab) showed significantly higher scores in the RMEAD, FSS and PHQ compared to negative controls.  A fatigue score of 36 or more, indicating clinically relevant fatigue, was obtained for 260 (10.6%) of SARS-CoV-2 negative participants, for 22 (10.0%) of seropositive participants without positive swab (OR 0.9, 95% CI 0.6-1.5, p=0.86) and for 130 (23.7%) of NPS positive participants (OR 2.6, 95% CI 2.1-3.3, p<0.001). |
| **Uvais 2022**  **(India)** | Cross-sectional questionnaire study of HCW who have previously tested positive for COVID-19. Chi^2^, t-tests and Pearson correlations used to test differences between groups  Follow-up: NR | Explore the prevalence of depression, anxiety and PTSD among HCW recovered from COVID-19 in South India | Total n=107  Occupation: NR  Age group, n (%): 20-30, 84 (78.5); 31-40, 19 (17.8); 41-50, 4 (3.7)  Sex: 68 female (63.6%), 39 male (36.4%) | 71.6% reported persistent symptoms: depression (26.2%), anxiety (12.2%) and PTSD (3.4%) |
| **D'Avila 2023 (Brazil)** | Prospective cohort study of HCWs with confirmed COVID-19. Categorical comparisons analysed with Pearson’s chi-squared test. Continuous variables compared with ANOVA  Follow-up: 6 months | Assess prevalence of symptoms, of post-COVID-19 syndrome and HRQoL among HCWs 6 months after severe acute COVID-19 | Total n=289 (n=174 at 6 weeks)  Occupation: NR  Age, mean (SD): 42.2 (9.5)  Sex: 56 male (19.4%), 233 female (80.6%) | 63/174 (36.2%) assessed at 6 months were diagnosed with post-COVID-19 syndrome. Most frequent symptoms were fatigue (23/63, 36.5%), sleep disturbance (9/63, 14.3%), dyspnoea (8/63, 12.7%) and cough (6/63, 9.5%). 74.1% of those with reduced QoL at 6 months were due to post-COVID syndrome |
| **Shukla 2023**  **(India)** | Cross-sectional study conducted in eight tertiary centres. Descriptive statistics, odds ratios with 95% confidence intervals, Chi^2^ and simple logistic regression to measure association between medical sequelae and risk factors. Subgroup analysis between HCWs who recovered within 12–24 weeks and HCWs who recovered between >24 and 52 weeks  Follow-up: 12-52 weeks | Explore the COVID-19 sequelae and associated risk factors among HCWs | Total n=679  Occupation: 270 doctors (39.8%), 181 nurses (26.7%), 228 paramedical and ancillary staff (33.6%)  Age group, n (%):  <45 years, 596 (87.8%)  >45 years, 83 (12.2%)  Sex: 345 female (50.8%), 334 male (49.2%) | Of the 206/679 (30.3%) HCWs who reported medical sequelae, 7.5% participants had 2 sequelae and 9.7% had 3 or more.  Most frequent sequelae were fatigue (11.5%) insomnia (8.5%). Other symptoms included: difficulty in breathing during physical activity (6%), joint pain (5%), muscle soreness (4.4%) and fever (2.8%), cough, headache, loss of smell (4.6% each respectively), loss of taste (4%), sore throat (1.9%), reduced appetite (1.3%) and skin rash (1%) |
| **Stepanek 2023 (Czech Republic)** | Cross-sectional study of HCWs with post-acute COVID-19 syndrome (PCS). Descriptive statistics, t-test, Mann-Whitney test or Chi^2^ to determine significance of differences; logistic regression to explore relationship between post-COVID syndrome and other variables  Follow-up: ≥12 weeks | Determine clinical and laboratory characteristics and predictors of PCS in affected HCWs based on a comparison with HCWs who did not develop PCS | Total n=305 (181 cases and 124 controls)  Occupation: NR  Age, Mean (95%CI): PCS, 47.3 (45.9, 48.8); controls, 42.4 (40.5, 44.3)  Sex: PCS, 156 female (86.2%), 25 male (13.8%); controls, 89 female (71.8%), 35 male (28.2%) | Mean number of PCS symptoms: 1.9. Most frequently reported symptoms: Fatigue (47.5%), shortness of breath (38.1%), aches (16%), loss of smell (14.9%), sleep disorder (11%), loss of taste (9.4%) |

Note. NR: not reported; UK: United Kingdom; IQR: interquartile range; y: Years; PCS: post-COVID-19 syndrome; HCW: healthcare workers; SD: standard deviation
