# Supplementary material for: What is the impact of long-term COVID-19 on workers in healthcare settings? A rapid systematic review of current evidence
Source: PLoS One. 2024 Mar 5;19(3):e0299743. doi: 10.1371/journal.pone.0299743 (PMC10914278; doi:10.1371/journal.pone.0299743)
Supplement: S3 Table — (DOCX) [file pone.0299743.s004.docx]

| **Study ID** | **Healthcare workers experiencing long-term symptoms, n/N (%)** | **Details of symptoms, n (%) unless specified** | |
| --- | --- | --- | --- |
| **Brandt 2021^17^** | 3/13 (23.1)  [6 months after infection] | - 1/13 (7.7%): Ongoing anosmia, ageusia and dysgeusia; fatigue after moderate physical activity - 1/13 (7.7%): Unilateral paraesthesia in the ophthalmic nerve area - 1/13 (7.7%): Intermittent weakness in one leg and numbness in the cheek | |
| **Gaber 2021^18^** | 61/138 (44.2)  [time point NR] | - 54/138 (39.1%): moderate-to-severe fatigue - 55/138 (39.9%): mild-to-moderate shortness of breath - 67/138 (48.6%): sleep disturbance - 61/138 (44.2%): mood disorders - 42/138 (30.4%): struggling to cope with symptoms - 3/11 (27.3%) males and 58/127 (45.7%) females: persistent symptoms | |
| **Havervall 2021^19^** | 84/323 (26.0)^a^  [≥2 months after infection]  48/323 (14.9)^a^  [≥8 months after infection] | - ≥1 symptom for ≥2mo: 84 (26.0%) - ≥1 symptom for ≥8mo: 48 (14.9%) - Symptoms lasting ≥2mo/8mo, n (%):   - Anosmia: 47 (14.6%)/ 29 (9.0%)   - Fatigue: 27 (8.4%)/ 13 (4.0%)   - Ageusia: 25 (7.7%)/ 12 (3.7%)   - Dyspnoea: 14 (4.3%)/ 6 (1.9%)   - Sleeping disorder: 10 (3.1%)/ 7 (2.2%)   - Headache: 9 (2.8%)/ 5 (1.5%)   - Palpitations: 8 (2.5%)/ 2 (0.6%)   - Concentration impairment: 7 (2.2%)/ 2 (0.6%)   - Muscle/joint pain: 6 (1.9%)/ 2 (0.6%)   - Memory impairment: 5 (1.5%)/ 1 (0.3%) | |
| **Martinez 2021^20^** | 69/260 (26.5%) [>3 months after infection] | 26.5% participants had not regained their usual level of health or had symptoms lasting >3 months. 45 participants reported details of symptoms.   - Fatigue: 68.9% - Impaired taste or smell: 51.1% - General weakness: 46.7% - Concentration problems: 44.4% - Breathing problems: 42.2% - Sleep difficulties: 28.9% - Headache: 22.2% - Dizziness: 22.2% - Chest pain: 20.0% - Muscle pain: 20.0% - Hair loss: 17.8% - Palpitations: 15.6% - Cough: 11.1% - Joint pain: 8.9% - Feverish feeling: 6.7% - Decreased appetite: 6.7% - Digestive problems: 4.4%   37 reported persisting symptoms over 365 days including 106 cumulative missed workdays; the most common reported symptoms among them were fatigue (5 participants, 100%), general weakness (4 participants, 80%) impaired sense of taste or smell and palpitations (3 participants, 60%).  32 patients who reported the diagnosis of SARS-CoV infection to have been made more than 365 days ago with a symptom duration of 365 days or less reported 303 cumulative missed workdays | |
| **Mattioli 2021^21^** | 78/120 (65%) [4 months after infection] | - Anosmia: 23 (19.2%) - Fatigue: 18 (15%) - Headache: 15 (12.5%) - Attention difficulties: 14 (11.7%) - Ageusia: 13 (10.8%) - Dyspnoea: 13 (10.8%) - Joint and muscle pain: 11 (9.2%) - Insomnia: 8 (6.7%) - Memory difficulties: 8 (6.7%) - Irritability/anxiety: 6 (5%) - Hair loss: 4 (3.3%) - Arrhythmias: 3 (2.5%) - Hearing loss: 2 (1.6%) - Tremor: 2 (1.7%) - Dizziness: 1 (0.8%) - Radicular pain: 1 (0.8%) - Cough: 1 (0.8%) - Neurological deficits: 2 (1.7%) - DASS-21 anxiety, median (range): 3 (0-18) - DASS-21 stress, median (range): 7 (0-32) - DASS-21 depression: 3 (0-30) | |
| **Nielsen 2021^22^** | Positive PCR/Negative PCR:  44.1%/20.2% [days 31-60]^b^  38.5%/14.7% [days 61-90]^b^ | - Positive PCR, days 31-60/61-90, % of daily recordings^b^   - Any symptom: 44.1%/38.5%   - Reduced or lost taste or smell: 29.3%/28.6%   - Dyspnoea: 4.7%/3.5%   - Headache: 8.8%/6.6%   - Cough: 10.6%/4.1%   - Sore throat: 3.0%/2.8%   - Muscle ache or pain: 3.4%/3.6%   - Fever: 0.1%/0.0% | - Negative PCR, days 31-60/61-90, % of daily recordings^b^    - Any symptom:   - Reduced or lost taste or smell: 1.7%/0.9%   - Dyspnoea: 1.0%/0.5%   - Headache: 7.9%/5.3%   - Cough: 7.9%/5.5%   - Sore throat: 5.1%/4.0%   - Muscle ache or pain: 2.4%/2.3%   - Fever: 0.1%/0.1% |
| **Pereira 2021^23^** | 21/38 (55.3)  [7-8 months after symptom onset] | - Fatigue: 12/21 (57%) - Loss of smell: 6/21 (29%) - Breathlessness: 5/21 (24%) - Difficulty concentrating: 5/21 (24%) - 8/21 (38%) had 1 symptom; 6/21 (28.6%) had 2 symptoms and 7/21 (33.3%) had ≥3 symptoms - Ongoing symptoms were more common in people of BAME origin (10/14) but the difference was NS - Ongoing symptoms were more common in females (63%) than males (17%) but the difference was NS | |
| **Rao 2021^24^** | NR | - Health issues in the post COVID-19 period:   - Fatigue on mild exertion: 42.9%   - Breathlessness: 8.6%   - Headaches, myalgia: 15.3%   - Fever, cough, sore throat: 5.5%   - Loss of taste, and smell: 21.5%   - Depression: 3.1%   - Anxiety: 6.1%   - None: 33.7% - Frequency of health issues in the post COVID-19 period:   - Almost daily: 28.2%   - 3-4 times/week: 18.6%   - Once a week: 9.7%   - Occasionally, maybe once in 2 weeks: 43.6% - Major concerns in the post COVID-19 period:   - Fear of contracting virus again: 46.5%   - Spreading to family members: 53.6%   - Developing post COVID-19 complications: 34.6%   - Being isolated socially: 16.4%   - Shortage of facilities: 5.7%   - Financial: 17.0% | |
| **Sultana 2021^25^** | 44/186 (23.7)  [>60 days since infection] | - 44/186 (23.7%) reported at least one long post-COVID symptom (i.e. >60 days) - 130/186 (69.9%) had at least one acute post-COVID symptom (up to 60 days) - Symptoms 31-60 days/>60 days, n (%):   - Difficulty breathing: 4 (2.2%)/12 (6.5%)   - Cough: 2 (1.1%)/0   - Palpitation: 2 (1.1%)/0   - Chest pain: 1 (0.5%)/1 (0.5%)   - Fatigue: 10 (5.4%)/15 (8.1%)   - Sleep disturbance: 1 (0.5%)/7 (3.8%)   - Lack of concentration: 3 (1.6%)/9 (4.8%)   - Memory lapses: 1 (0.5%)/8 (4.3%)   - Headache: 3 (1.6%)/1 (0.5%)   - Anosmia: 4 (2.2%)/0   - Irritability: 0/2 (1.1%)   - Loss of taste: 1 (0.5%)/1 (0.5%)   - Anxiety: 0/1 (0.5%)   - Loss of appetite: 0/1 (0.5%)   - Nausea: 1 (0.5%)/0   - Joint pain: 0/3 (1.6%)   - Hair fall: 0/8 (4.3%) | |
| **Tawfik 2021^26^** | NR | ^c^Five most commonly reported symptoms at 1 month   - Fatigue: 75% - Dyspnoea: 50% - Depressive symptoms: 50% - Headache: 42% - Myalgia: 40%   ^c^Five most commonly reported symptoms at 3 months   - Fatigue: 33% - Dyspnoea: 29% - Depressive symptoms: 20% - Headache: 19% - Bony aches: 18% | |
| **Tempany 2021^27^** | 98/139 (70.5%)^d^  [≥12 weeks since infection] | 98/139 (70.5%) reported persistent symptoms, n (%):   - Fatigue: 78 (56.1%) - Sleep disturbance: 56 (40.3%) - Cognitive impairment: 34 (24.5%) - Psychological symptoms: 30 (21.6%) - Other physical symptoms: 30 (21.6%) | |
| **Akova 2022^28^** | 133/133 (100%)  [HCW with Long COVID recruited] | 74/133 (55.6%) were fatigued/over-fatigued (i.e. Fatigue Assessment Scale score ≥22)  79/133 (59.4%) reported poor sleep quality (i.e. Pittsburgh Sleep Quality Index score ≥5) | |
| **Carazo 2022^29^** | Non-hospitalised HCW: 46.2% with symptoms≥4 weeks, 39.9% ≥12 weeks  Hospitalised HCW: 76.3% with symptoms≥4 weeks, 67.6% ≥12 weeks | Hospitalised vs non-hospitalised HCWs with symptoms lasting ≥4 weeks:   - Fatigue: 30% vs 64% - Loss of smell/taste: 20% vs 17% - Shortness of breath: 20% vs 56% - Cognitive dysfunction: 15% vs 33% - Headache: 13% vs 23% - Joint & muscular pain: 10% vs 22% | |
| **Kameyama 2022^30^** | 60/83 (72.2%) at 1 month; 32/83 (38.6%) at 3 months; 17/83 (29.5%) at 6 months after infection | Most common symptoms at 1 month:   - Anosmia: 33.7% - Fatigue: 33.7%   At 3 months:   - Anosmia: 18.1% - Fatigue: 9.6%   At 6 months:   - Anosmia: 7.2% - Fatigue: 4.8%   Median EQ-VAS score: 75.0  Median motivation for continuing to work score: 4 (0=no motivation, 10=maximum motivation) | |
| **Kaplan 2022^31^** | 77/121 (63.6%) at >3 weeks after COVID-19 infection; 38/121 (31.4%) at >12 weeks after infection; 19/121 (24.6%) at >24 weeks after infection | Symptoms lasting > 3 weeks (n, %):  Fatigue (40, 33%), loss of smell (27, 22.3%), attention deficit/concentration disorder (25, 20.7%), dyspnoea (24, 19.8%), myalgia (24, 19.8%), loss of taste (23, 19%), cough (19, 15.7%), joint pain (18, 14.9%), sleep disturbance (14,11.6%), and memory difficulties (13, 10.7%)  Symptoms lasting >12 weeks (n, %):  Loss of smell (16, 13.2%), loss of taste (11, 9.1%), fatigue (10, 8.6%), attention deficit and concentration disorder (9, 7.4%), dyspnoea (8, 6.6%), sleep disturbance (7, 5.7%), cough (5, 4.1%), chest pain (4, 3.3%), memory difficulties (4, 3.3%), headache (3, 2.4%), myalgia (3, 2.4%), joint pain (1, 0.8%), sputum (1, 0.8%), constipation (1, 0.8%), and back pain (1, 0.8%)  Symptoms lasting >24 weeks (n, %):  Loss of smell (9, 11.6%), loss of taste (5, 6.4%), dyspnoea (5, 6.4%), headache (3, 3.8%), fatigue (2, 2.5%), cough (2, 2.5%), attention deficit and concentration disorder (2, 2.5%), memory difficulties (1, 1.2%), sleep disorder (1, 1.2%), back pain (1, 1.2%) | |
| **Kinge 2022^32^** | 15/62 (24.2%) [symptoms experienced for ≥3 months] | - Persistent COVID-19 symptoms at three months and longer: 15 (24.2%) - 33% of those with persistent symptoms reported more than one persistent symptom   Most commonly reported post-acute COVID-19 symptoms [timepoint NR]:   - Fatigue: 42% - Anxiety: 34% - Difficulty sleeping: 31% - Chest pain: 24% - Brain fog: 21% - Muscle pain: 21% - Joint pain: 18% | |
| **Mendola 2022^33^** | NR [questionnaire completed at mean 18 months since acute infection] | Post-COVID-19 symptoms among HCWs hospitalised due to COVID-19:   - Cough: 30 (57%) - Resting dyspnoea: 33 (62%) - Exertional dyspnoea: 46 (87%) - Arthromyalgia: 38 (72%) - Chest pain: 17 (32%) - Tachycardia or palpitations: 19 (36%) - Ageusia: 23 (43%) - Anosmia: 25 (47%) - Asthenia: 46 (87%) - Cephalgia: 25 (47%) - Loss of memory: 25 (47%) - Hair loss: 22 (41%) - Sleep disorders: 34 (64%) - Anxiety/depression: 25 (47%) | |
| **Mohr 2022^34^** | 298/419 (71%)  [6 weeks after illness onset] | ^c^Prevalence of symptoms at 6 weeks after COVID-19 symptom onset  [Vaccinated (n=180) / unvaccinated (n=239):   - Fatigue: 35% / 48% - Dyspnoea: 15% / 30% - Cough: 25% / 30% - Sinus congestion: 25% / 30% - Myalgia: 15% / 25% - Nausea: <5% / 10% - Diarrhoea: 5% / 8% - Sore throat: 8% /8 % - Chills: 0 / 5% - Vomiting: 0 / 2% - Fever: 0 / 1% - Loss of taste or smell: 22% / 35% - Headache: 20% / 30% - Concentration problems: 25% / 25% - Memory difficulties: 20% / 22% - Dizziness: 10% /15% - Confusion: 4% / 5% - Movement disorders: <5% / <5% - Trouble sleeping: 22% / 30% - Exercise problems: 22% / 28% - Chest pain: 6% / 10% - Abdominal pain: <5% / <5% | |
| **Nehme 2022^35^** | NR | Median time from infection to follow-up was 244 days (interquartile range IQR 202–400 days) in HCWs.  Presence of symptoms in COVID-19 positive HCWs vs negative HCWs, aOR (95%CI):   - Loss or change in smell: 11.79 (6.29, 22.09), p<0.001 - Loss or change in taste: 11.58 (5.23, 25.64), p<0.001 - Palpitations: 7.27 (2.09, 25.29), p=0.002 - Dyspnoea: 3.71 (2.06, 6.70), p<0.001 - Difficulty concentrating/memory loss: 2.00 (1.30, 3.09), p=0.002 - Fatigue: 1.59 (1.23, 2.06), p<0.001 - Headache: 1.60 (1.09, 2.34), p=0.017 - Myalgia: 1.47 (0.92, 2.36), p=0.109 - Arthralgia: 1.50 (0.92, 2.44), p=0.102 - Cough: 1.60 (0.77, 3.32), p=0.207 - Chest pain: 1.15 (0.36, 3.62), p=0.811 - Exhaustion/burnout: 1.51 (0.92, 2.47), p=0.100 - Insomnia: 1.26 (0.81, 1.97), p=0.300 - Stress: 0.59 (0.30, 1.19), p=0.141 | |
| **Otmani 2022^36^** | 56/118 (47.4%) [timepoint NR] | 56/118 (47.4%) experienced at least one symptom of Long COVID:   - Anosmia/hyposmia: 9.6% - Dysgeusia: 6% - Tinnitus: 7.2% - Dyspnoea: 3.6% - Cough: 4.8% - Chest pain: 8.4% - Palpitations: 10.8% - Myalgia: 13.3% - Arthralgia: 9.6% - Abdominal pain: 4.8% - Diarrhoea: 6% - Itching: 1.2% - Headache: 12% - Dizziness: 8.4% - Sensitive disorders: 1.2% - Sleep disorders: 12% - Anxiety: 21.7% - Attention disorders, memory impairment, brain fog: 14.4% | |
| **Pilmis 2022^37^** | 24/74 (32.4%) [7-month cohort study] | - Asthenia: 12 (16.2%) - Dyspnea: 10 (13.5%) - Concentration disorder: 7 (9.5%) | |
| **Selvaskandan 2022^38^** | 43/120 (36%) [beyond 3 months after infection] | - Fatigue: 30 (70%) - Mood changes: 8 (19%) - Ageusia/anosmia: 6 (14%) | |
| **Senjam 2022^39^** | 156/395 (39.5%) | 39.5% of hospital employees reported post-COVID symptoms at ≥4 weeks after infection. The multivariable regression analysis showed that non-healthcare staff were at lower risk of having post-COVID symptoms than employees working in the hospital (OR: 0.65, 95%CI 0.74-3.87) | |
| **Strahm 2022^41^** | Proportion of HCWs reporting one or more symptoms compatible with Long COVID:   - Positive nasopharyngeal swab (NPS): 73% - Only seropositive: 58% - Negative controls: 52% | The most common symptoms were exhaustion/burnout (33% in NPS-positive vs. 25% in only seropositive vs. 24% in negative controls) and weakness/tiredness (34% vs. 25% vs. 22%). Impaired taste/olfaction (33% vs. 16% vs. 6%) and hair loss (17% vs. 17% vs. 10%) were the only symptoms which were significantly more common in only seropositive HCW compared to negative controls | |
| **Uvais 2022^42^** | 73/102 (71.6) [timepoint NR] | 73/102 (71.6%) reported persistent symptoms   - Depression: 26.2% - Anxiety: 12.1% - PTSD: 3.7% | |
| **D’Avila 2023^43^** | 63/174 (36.2%) [6 months after acute COVID-19 infection] | 63/174 (36.2%) diagnosed with post-COVID-19 syndrome:   - Fatigue: 23/63 (36.5%) - Sleep disturbance: 9/63 (14.3%) - Dyspnoea: 8/63 (12.7%) - Cough: 6/63 (9.5%) - Reduced QoL due to post-COVID-19 syndrome: 63/85 (74.1%) | |
| **Shukla 2023^40^** | 206/679 (30.3%) [between 12-52 weeks after COVID infection] | - Fatigue: 78 (11.5%) - Pain in joints: 34 (5%) - Soreness in muscles: 30 (4.4%) - Fever: 19 (2.8%) - Difficulty in breathing during physical activity: 41 (6%) - Cough: 31 (4.6%) - Tightness in chest: 15 (2.2%) - Throat Pain: 14 (2%) - Difficulty in breathing while at rest: 11 (1.6%) - Sensation of irregular or fast heartbeat: 8 (1.2%) - Reduced Appetite: 9 (1.3%) - Nausea: 7 (1%) - Diarrhoea: 6 (0.9%) - Abdominal pain: 4 (0.6%) - Sore throat: 13 (2%) - Pain in the ear: 3 (0.4%) - Ringing sensation in ears: 2 (0.3%) - Headache: 31 (4.6%) - Loss of smell: 31 (4.6%) - Loss of taste: 27 (4%) - Difficulty in concentrating: 14 (2%) - Difficulty to focus on the usual things: 12 (1.8%) - Forgetting things easily: 11 (1.6%) - Pins & needles sensation or numbness in hands or feet: 7 (1%) - Difficulty in thinking clearly or getting anything done: 5 (0.7%) - Sleep disorder (Insomnia): 58 (8.5%) - Depression: 9 (1.3%) - Stress: 7 (1%) - Anxiety: 1 (0.2%) - Skin rash: 7 (1%)   COVID sequelae were significantly higher among HCWs ≥ 45 years of age (OR 2.03; 95% CI 1.27–3.25) and those with comorbidity (OR 2.01). In contrast, the odds of having sequelae were found to be significantly lesser among males (OR 0.55) and among doctors as well as doctors and nursing staff as a combined group compared to other HCWs (OR 0.65 and 0.70, respectively). Logistic regression analyses confirmed that moderate-severe COVID was an independent predictor for risk of having COVID sequelae (adjusted OR 5.83; 95% CI 3.05–11.14) and male gender was a protective factor (adjusted OR 0.56; 95% CI 0.4–0.8) | |
| **Stepanek 2023^44^** | 181/305 (59.3) [≥12 weeks after acute infection] | - Mean number of PCS symptoms: 1.9 (median 2) - Persisting tiredness or fatigue that interfered with daily life: 86 (47.5%) - Shortness of breath: 69 (38.1%) - Muscle, joint or body aches: 29 (16%) - Loss of smell: 27 (14.9%) - Headache: 27 (14.9%) - Sleep disorder: 20 (11%) - Loss of taste: 17 (9.4%) - Cough: 16 (8.8%) - Chest pain or pressure: 14 (7.7%) - Hair loss or skin problems: 8 (4.4%) - Depression or anxiety: 5 (2.8%) - Palpitations: 4 (2.2%) - Rash: 4 (2.2%) - Visual impairment: 3 (1.7%) - ‘Brain fog’: 2 (1.1%) - Following symptoms reported by one participant (0.6%) each: runny nose, fever, pins-and-needles, diarrhoea, sweating and speech disorders | |
