# Supplementary material for: What is the impact of long-term COVID-19 on workers in healthcare settings? A rapid systematic review of current evidence
Source: PLoS One. 2024 Mar 5;19(3):e0299743. doi: 10.1371/journal.pone.0299743 (PMC10914278; doi:10.1371/journal.pone.0299743)
Supplement: Attachment — Submitted filename: Response to reviewers.docx [file pone.0299743.s005.docx]

The Editor

23rd November 2023

Dear Editor

# What is the impact of long-term COVID-19 on workers in healthcare settings? A rapid systematic review of current evidence

This review was previously submitted to PLOS ONE in May 2023 and we hereby submit a revised version of the manuscript addressing the points raised by the reviewers to whom we are most grateful for their valuable points about our work. Please see the table below for our responses.

| **Reviewer comment** | **Author response** |
| --- | --- |
| **REVIEWER 1** | |
| 1. ABSTRACT: The abstract should be structured into Background, Objective, Methods, Results and Conclusion.  *Include a brief background for the study *Discussion is usually not part of abstract *The result section of the abstract should be shortened /summarized to highlight only the major and important findings. | Thank you for your comment. The Abstract has been restructured as suggested. |
| 2. Line 43-- “Rationale” should be deleted since the study has been justified in the latter part of the introduction | Text deleted as suggested. |
| 3. Line 91—“Objectives” should be deleted, lines 92-94 should be moved to the introduction as an aim of the study | Text amended as suggested. |
| 4. Tables 1 and 2 appear quite complex and ambiguous. I suggest these 2 tables should be simplified and summarized to highlight and showcase the important and pertinent findings of the previous works done. Information like methods of data analysis and study objectives may be left out while information on participants’ characteristics and sample size should be summarized. What is more important is the summary of main findings. | Thank you for your suggestion. Table 1 has been deleted and some clarifying text has been added in its place. Tables 2 and 4 have been amalgamated and simplified, with the findings being the main focus. |
| 5. Line 403 “Results of quantitative studies” --- This seems like a repetition of table 2. Kindly summarise or state what is not obvious from table 2 | The section has been shortened, whilst retaining important information about proportions of participants experiencing symptoms and respective timeframes. |
| 6. Table 4 seems more like a repetition of table 2. | The two tables have now been amalgamated. |
| 7. Line 474 “Summary of findings” should be removed | Text deleted as suggested. |
| 8. Was there any difference in Long covid symptoms between males and females? | Comparing symptoms between males and females was not an objective of our review and most studies did not report results for males and females separately. We acknowledge the potential importance of this comparison and have added a recommendation for future research. |
| 9. There should be a conclusion paragraph in the discussion which should summarise the findings of this study. | A conclusion paragraph has been added. |
| **REVIEWER 2** | |
| 1. Please was this review registered? Please quote the registration number. | The review protocol was registered on the PROSPERO database (CRD42021288181), as stated in the Methods section of the manuscript. |
| 2. ‘Discussion’ is NOT classically part of abstract. The abstract should highlight aim, methods, results, limitations and strength and conclusions from the work. | Thank you. The Abstract has been revised accordingly. |
| 3. Could this be a limitation? Your work did not gather information that relates Long Covid with covid vaccination. Could those symptoms follow effect of vaccination or Long Covid? | Thank you for pointing this out. Indeed, our study did not examine the association between vaccination and Long COVID and we have added this as a limitation and a recommendation for future research. |
| 4. Authors should please include the keywords used with extensions in their search strategy. Please revisit appendix 1 for a clearer search strategy and keyword representation. | The search construction, text terms, and syntax have been copied directly from Medline; they are in a format that will allow the search to be replicated should a reader wish to do so. |
| 5. Please what tool / software was used for data screening, and extraction? Please indicate. | No tools or software were used. Data screening and extraction were carried out by the review authors as detailed in the Methods section of the manuscript. |
| 6. “There was no restriction on language or study type at the search stage. Results were limited to those published from December 2019. Searches were all carried out in November 2021 and updated in December 2022.” Please did you exclude 2020 period in this study? See Line 135, 136, 137. | There was no restriction on language, study type, or date at the search stage. The final searches were carried out in December 2022. |
| 7. What tool was used in the assessment of list of Bias? Line 236 | Risk of bias was assessed using the Quality of Reporting Tool (QuaRT), as reported in the Methods section of the manuscript. |
| 8. I suggest that the discussion section be shortened by half as this segment appear verbose. | The Discussion section has been shortened. |
| 9. A sub-analysis of long COVID versus covid vaccination was not considered in this study. Can this be a Limitation? This study was limited to hospital only, could this be a limitation? | Please see point 3 above regarding the vaccination versus Long COVID association. Our review was not limited to hospital staff. All clinical and non-clinical staff were eligible, in any relevant settings, except for care homes and other long-term care facilities. |
| 10. Someone proficient in English to go through the whole manuscript correcting all syntax and typographical errors e.g. “The medicolegal aspect is huge, and I think possibly certainly feels that way as a GP and it’s scary to not be able to recognize potentially where you have deficits because if you can’t recognize them then that’s an UNKNOWN UNKNOWN in what can you do with that. And just the sort of fast-paced nature of GP and as…” See line 267. Repeated word highlighted. | Thank you for pointing this out. The text highlighted by the reviewer is one of several quotes that are reported verbatim from the included studies. Please note that people’s quotes are never ‘corrected’ in qualitative research. Quotes reflect what the people have said during their interviews with the authors of the included studies and we have no control of them. We trust that using italics for verbatim quotes and plain text for our own words makes the distinction clear for readers. |
| 11. I am not comfortable with the placement of tables in between text. International manuscript standards prefer tables and figures after the reference lists. | Tables and Figures have been moved to after the reference list and placeholders inserted in the text. |
| 12. Authors should write in such a manner as to avoid plagiarism | Please note that by its nature, a review including qualitative research reports verbatim material. This is explicitly stated in the methods and all material is attributed to its original source; therefore, there is no plagiarism issues that need to be addressed. |
| **REVIEWER 3** | |
| Basically the writing is very good, but should be given a year limit, wheater the last 3 years so that the data is more relevant. Though there are grammatical and editorial problem that need correction in ur papaer. | Thank you for your comment. Regarding the relevance of the data, our review includes literature from 2019 to 2022, so covers the period of three years from the onset of the pandemic. Thus, the data are considered relevant.  We believe that the grammatical issues the reviewer is referring to relate to the content of quotations taken from the included studies. As explained above, we have no control over the content of these quotations, which reflect what the interviewed people actually said. It is the content rather than the appropriateness of the language itself that is of interest here. Moreover, verbatim quotes (including errors) can provide information on the linguistic competence or emotions of the people interviewed. All editorial issues have been addressed. |

We look forward to receiving your response.

Yours sincerely,

Dr Moira Cruickshank

mcruickshank@abdn.ac.uk
