# Supplementary material for: What is the impact of long-term COVID-19 on workers in healthcare settings? A rapid systematic review of current evidence
Source: PLoS One. 2024 Mar 5;19(3):e0299743. doi: 10.1371/journal.pone.0299743 (PMC10914278; doi:10.1371/journal.pone.0299743)
Supplement: Attachment — Submitted filename: Response to reviewers.docx [file pone.0299743.s006.docx]

Dr Chenette

Editor-in-Chief

PLOS ONE

(via electronic submission system)

2^nd^ February 2024

Dear Dr Chenette

# Manuscript: What is the impact of long-term COVID-19 on workers in healthcare settings? A rapid systematic review of current evidence

This manuscript was submitted to PLOS ONE on 23^rd^ November 2023 and we hereby submit a revised version of the manuscript addressing the points raised by the Editor. The table below details our responses.

| **Editor’s comment** | **Authors’ response** |
| --- | --- |
| The Introduction should be abbreviated to 300-400 words maximum | The Introduction has been shortened accordingly |
| The methodology should be made more concise and precise leaving out unnecessary details. | The Methods section has been revised to focus on pertinent details |
| Results of qualitative studies: Reduce quotes to a maximum of 2 salient and relevant quotes under each theme | A maximum of two quotes have been provided under each theme |
| Results of quantitative results: Listing every symptom and the number of studies where each was found is unnecessary. List the most important/most common symptoms and other symptoms can be mentioned collectively with appropriate references. | These results have been revised accordingly |
| The discussion should be abbreviated to 500-600 words maximum. The Discussion should explain the study findings with relevant literature references/citations and also compare with similar or previous studies. | The Discussion has been shortened and accordingly |

We look forward to receiving your response.

Yours sincerely,

Dr Moira Cruickshank

mcruickshank@abdn.ac.uk
